# The T850D Phosphomimetic Mutation in the Androgen Receptor Ligand Binding Domain Enhances Recruitment at Activation Function 2

**DOI:** 10.3390/ijms23031557

**Published:** 2022-01-29

**Authors:** Christine Helsen, Tien Nguyen, Thomas Vercruysse, Staf Wouters, Dirk Daelemans, Arnout Voet, Frank Claessens

**Affiliations:** 1 Laboratory of Molecular Endocrinology, Department of Cellular and Molecular Medicine, KU Leuven, ON I, 3000 Leuven, Belgium; frank.claessens@kuleuven.be; 2 Laboratory of Biomolecular Modelling and Design, Department of Chemistry, KU Leuven, Celestijnenlaan 200G, 3001 Leuven, Belgium; truongtien.nguyen@kuleuven.be (T.N.); staf.wouters@kuleuven.be (S.W.); arnout.voet@kuleuven.be (A.V.); 3 Laboratory of Virology and Chemotherapy, Department of Microbiology, Immunology and Transplantation, Rega Institute, KU Leuven, 3000 Leuven, Belgium; thomas.vercruysse@kuleuven.be (T.V.); dirk.daelemans@kuleuven.be (D.D.)

**Keywords:** androgen receptor, post-translational modification, allosteric regulation, molecular dynamics, gain of function, Helix 10, phosphomimetic

## Abstract

Several key functions of the androgen receptor (AR) such as hormone recognition and co-regulator recruitment converge in the ligand binding domain (LBD). Loss- or gain-of-function of the AR contributes to pathologies such as the androgen insensitivity syndrome and prostate cancer. Here, we describe a gain-of-function mutation of the surface-exposed threonine at position 850, located at the amino-terminus of Helix 10 (H10) in the AR LBD. Since T850 phosphorylation was reported to affect AR function, we created the phosphomimetic mutation T850D. The AR T850D variant has a 1.5- to 2-fold increased transcriptional activity with no effect on ligand affinity. In the androgen responsive LNCaP cell line grown in medium with low androgen levels, we observed a growth advantage for cells in which the endogenous AR was replaced by AR T850D. Despite the distance to the AF2 site, the AR T850D LBD displayed an increased affinity for coactivator peptides as well as the ^23^FQNLF^27^ motif of AR itself. Molecular Dynamics simulations confirm allosteric transmission of the T850D mutation towards the AF2 site via extended hydrogen bond formation between coactivator peptide and AF2 site. This mechanistic study thus confirms the gain-of-function character of T850D and T850 phosphorylation for AR activity and reveals details of the allosteric communications within the LBD.

## 1. Introduction

The androgen receptor (AR) is a ligand-activated transcription factor that belongs to the steroid receptor family [1]. Similar to other steroid receptors, it contains three domains: the amino-terminal domain (NTD), the DNA binding domain (DBD) and the ligand binding domain (LBD) [2]. Each domain has specific functions but communication as well as dimerization between different domains are required for full receptor activity [2,3,4,5].

The ligand-binding domain (LBD) is built up by 11 α-helical segments that form a hydrophobic pocket which can accommodate testosterone and dihydrotestosterone but also steroidal and non-steroidal AR (ant)agonists [6,7,8,9]. The LBD has docking sites (such as AF2 and BF3) on its surface for interaction with co-regulators and co-chaperones as well as a dimerization surface for a second AR-LBD [10,11,12,13]. Additionally, AF2 can engage in the so-called N/C interaction with the ^23^FQNLF^27^ motif of the NTD which has been proposed to stabilize hormone binding but also to affect co-regulator interactions [14,15].

The AR is mainly involved in the development and homeostasis of male reproductive organs but affects also other systems like the musculoskeletal system and the central nervous system [16,17,18,19,20]. Due to its location on the X chromosome, the AR is a hemizygous gene and is prone to disease-causing mutations. Germline mutations can lead to the androgen insensitivity syndrome while somatic mutations often arise as a response to hormone therapy for prostate cancer (PCa), thus leading to resistance to AR-targeting therapies [21,22]. The latter so-called gain-of-function mutations span the complete coding sequence of the AR with the majority of them present in the LBD (45%) [23]. In addition to such point mutations, other mechanisms that restore AR signaling during PCa progression have been well described. Among them are AR gene amplification, AR gene rearrangements, alternative splicing and changes in post-translational modifications such as AR phosphorylation [24,25].

While there are 13 AR phosphorylation sites with an identified function, only two of them, S791 and T850, are located in the LBD [26]. Due to its location close to the LBD dimerization surface, T850 was selected as the residue of interest in this study. T850 is located at the beginning of H10 and is a residue known to be phosphorylated by the PIM1 isoform, PIM1L, leading to AR stabilization [27].

Together with PIM2 and PIM3, PIM1 belongs to the PIM family of which the members have overlapping functions and comparable substrate recognition sequences [28,29]. PIM1 upregulation was found in different types of cancer as well as in PCa [30]. PIM1 has been implicated in prostate tumorigenesis, in resistance to both hormone therapy and chemotherapeutics [30,31,32,33]. While a regulatory role of AR by PIM1 is suggested to play a role in PCa, PIM1 has many downstream target proteins which could contribute to tumorigenesis. The T850D phosphomimetic mutation, representative for T850 phosphorylation by PIM1L, demonstrated a clear gain-of-function character which we explored further mechanistically in this study.

## 2. Results

### 2.1. T850D Increases AR Activity

**Figure 1:**
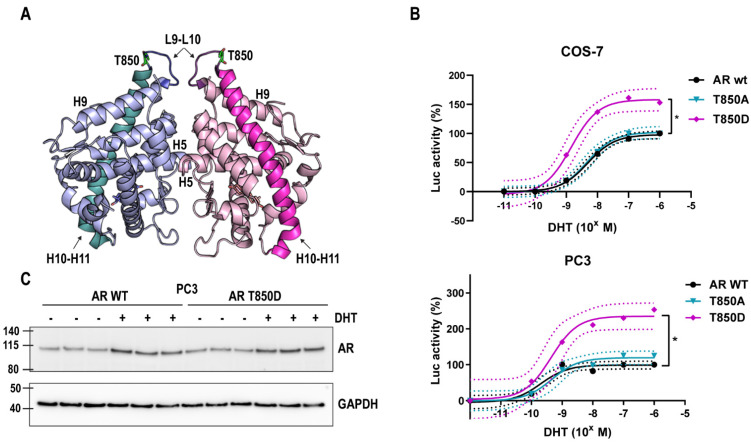
T850D increases AR activity. (**A**) T850 is located in the AR LBD in L9-L10 where it precedes the start of H10. H10 and H11 are an extended alpha helix that covers the ligand binding pocket (LBP). Two LBDs in dimeric configuration are shown, with H10-11 highlighted in cyan and in pink in their respective monomer. DHT is depicted in each LBP. Numbering of the helices is according to the 12-helical canonical fold of NRs but helix 2 is lacking in AR LBD. Adapted from PDB ID 5JJM using Pymol [34]. (**B**) Transcriptional activity of AR WT, AR T850A and AR T850D in COS-7 and PC3 cells. Mean values with 95% CI are given for each fitted curve (*n* ≥ 4). * = *p* < 005 (**C**) Androgens induce stability of AR WT and AR T850D. DHT (10 nM) was added to transfected PC3 cells for 2 h. AR levels were determined by Western Blot. GAPDH is shown as reference protein.

To determine the effect of T850D in AR transactivation, we introduced the phosphomimetic threonine (T) to aspartate (D) mutation in the full-size receptor and analyzed its activity in luciferase reporter assays (Figure 1). We observed that the transcriptional activation by AR T850D is higher than the activity of AR WT at all DHT concentrations. In COS-7 cells, this leads to a 60% higher maximal activity (*p* < 0.05) as well as a shift of the EC50 of AR WT vs. AR T850D from 4.89 nM to 1.47 nM (*p* < 0.05) (Figure 1B, Table S1). Similar findings of AR gain of function through T850D were observed in PC3 cells where the maximal activity of AR increased more than 2-fold (Figure 1B, Table S1). Western blot analysis showed that this increase of maximal activity was not correlated with an increased receptor level and that DHT led to a similar ligand-induced protein stabilization of the AR T850D compared to AR WT (Figure 1C).

### 2.2. Relevance of AR T850D in PCa Cells

**Figure 2:**
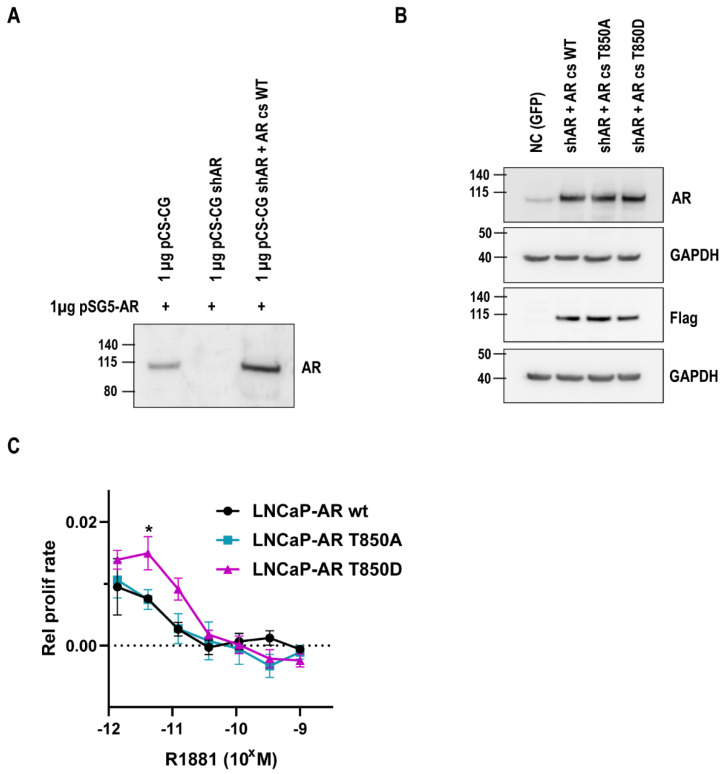
Effect of the AR T850D mutation on LNCaP proliferation. (**A**) Validation of lentiviral plasmids for AR replacement in HEK 293-T. Equal amounts (1 µg) of an AR-expression plasmid (pSG5-AR) and the lentiviral plasmid were transfected in HEK 293-T cells. Via WB, AR levels were detected for each condition: the empty lentiviral plasmid (lane 1), the shAR-expressing plasmid (lane 2) and the plasmid encoding the shAR and the codon-switched AR WT (lane 3). (**B**) Western blot of lentiviral LNCaP cell lines showing expression of flag-tagged codon-switched AR WT/T850A/T850D. The negative control (NC) was transduced with empty pCS-CG leading to the integration of a GFP expression cassette. GAPDH is shown as reference protein. (**C**) Proliferation of GFP-positive LNCaP cells in presence of R1881 was followed up for each lentiviral LNCaP cell line. Mean values with SEM are given for each data point (*n* = 3). An Ordinary one-way ANOVA with Dunnett’s multiple comparisons test was performed with AR WT as the control. * = *p* < 0.05.

In order to elucidate the potential role of T850D in PCa progression, we generated lentiviral LNCaP sublines that express a flag-tagged codon-switched AR to replace the endogenous AR T877A variant. The lentiviral plasmid contains a shRNA against endogenous AR and a replacement cassette encoding the codon-switched AR WT. The functionality of both components was validated via a transient co-transfection with the pSG5-hAR expression plasmid in HEK 293T cells (Figure 2A) and on the endogenous AR in LNCaP cells (Figure S7). Lentiviral transduction of LNCaP cells resulted in LNCaP sublines that express AR WT, AR T850D or AR T850A at comparable levels which can be observed in Figure 2B. Proliferation of these lentiviral LNCaP cell lines (AR WT, AR T850A and AR T850D) was compared in a R1881 concentration gradient. This revealed that LNCaP cells that express AR T850D proliferate better at low R1881 levels when compared to cells expressing AR WT and AR T850A (Figure 2C). The effect is most visible at 0.004 to 0.01 nM which correlates with androgen levels under castrate conditions.

### 2.3. AR Gain-of-Function by T850D Is Independent from the Ubiquitination Sites K845 and K847

**Figure 3:**
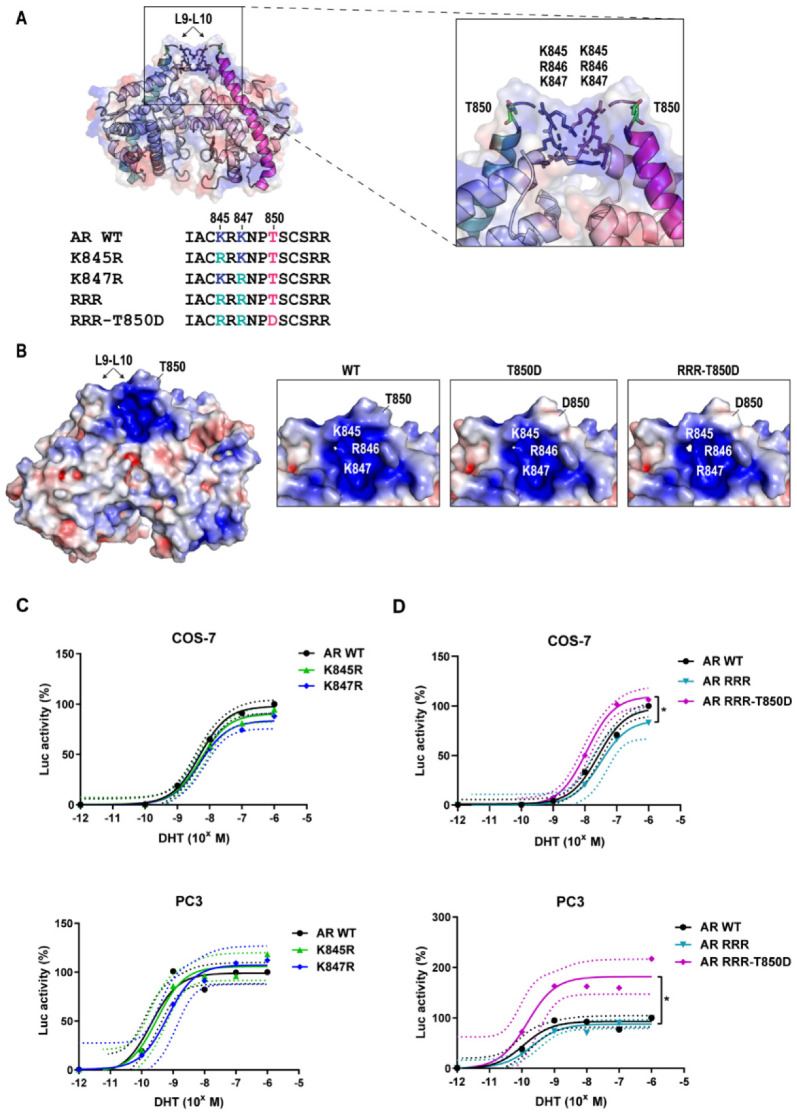
T850D and loss of ubiquitination at K845 and K847. (**A**) Visual representation of the localization of K845, R846, K847 and T850 within the amino acid sequence of AR 842–855 and within a 3D model of the AR LBD dimer. K845, R846, K847 and T850 are located in L9–L10. Adapted from PDB ID 5JJM. (**B**) Protein contact potential of the AR LBD dimer, which is a qualitative electrostatic representation with positive charges in blue and negative charges in red. From left to right: AR WT LBD dimer, close-up of WT L9-L10, T850D L9-L10 and AR RRR-T850D L9-L10. Mutation of K845 and K847 to R results in loss of the candidate ubiquitination sites but has no effect on the protein contact potential of the AR LBD dimer. Adapted from PDB ID 5JJM using the APBS Plugin for Pymol [34]. (**C**,**D**) Activity of AR WT and effect of K-to-R mutations in combination with the T850D mutation in COS-7 and PC3 cells. (**C**) K845R and K847R single mutants. (**D**) K845R-K847R double mutant combined (RRR) with T850D. Mean values with 95% CI are given for each fitted curve (*n* ≥ 4). * = *p* < 0.05.

In our search for the mechanism behind the AR gain-of-function by T850D, we explored the contribution of two nearby ubiquitination sites, K845 and K847. Linn et al. reported that RNF6 is recruited after phosphorylation of AR T850 and the consequent ubiquitination of K845 and K847 by RNF6 leads to a higher transcriptional activity of AR [27,35]. When mutating these ubiquitination sites close to T850, lysine (K) to arginine (R) mutations were chosen to retain the positive charge but to lose the ability to be ubiquitinated (Figure 3A,B). In our AR transactivation studies, we observed that the single and double mutations K845R or K847R did not significantly affect AR activity (*p* > 0.05) (Figure 3C, Table S1). However, the T850D mutation still increased the overall activity of the AR RRR, independent of these K to R mutations (Figure 3D, Table S1). For COS-7 cells, the maximal AR activity of AR RRR-T850D increased with 24% compared to AR RRR (*p* < 0.05), while in PC3 an increase of 94% was found (*p* < 0.05). This gain-of-function is not explained by a difference in AR levels (Figure S1). We can therefore conclude that the ubiquitination sites K845 and K847 do not contribute to the gain-of-function effect of T850D on AR.

### 2.4. Is T850D Part of the LBD Dimerization Interface?

**Figure 4:**
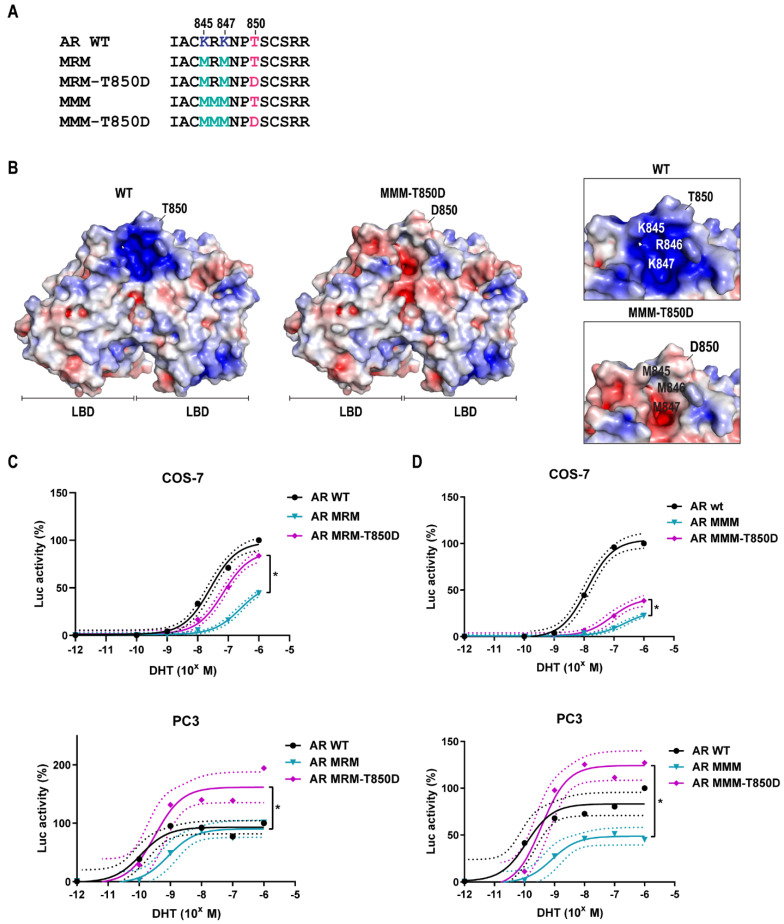
T850D and the LBD dimerization surface. (**A**) Amino acid sequence of AR 842–858. Mutation of K845, R846 and K847 to M results in loss of positive charges. (**B**) Electrostatic representation of the LBD dimer surface of AR WT and AR MMM T850D shows loss of positive charges by K845M, K846M and K847M. Close-up of WT L9-L10 vs. MMM-T850D L9-L10. Adapted from PDB ID 5JJM with the APBS Plugin for Pymol. (**C**,**D**) Activity of AR WT and effect of K/R-to-M mutations in combination with the T850D mutation in COS-7 and PC3 cells. (**C**) AR K845M-K847M double mutant (MRM). (**D**) AR K845M-R846M-K847M triple mutant (MMM). Mean values with 95% CI are given for each fitted curve (*n* ≥ 3). * = *p* < 0.05.

In the dimeric configuration of the AR LBD, T850 is surface exposed and close to a positively charged region on the surface of the dimeric partner (Figure 4A,B). This positive patch is made up by K845, R846 and K847 in L9-L10 (linker region between H9 and H10). The T850D mutation or phosphorylation of T850 introduces a negative charge which potentially takes part in an electrostatic interaction with this positive patch and as a consequence promotes LBD dimerization. We investigated this through the mutation of the positive region across the interface and the effect of it on the transcriptional activity of AR and AR T850D. The loss of positive charge induced by mutation of K845, R846 or K847 into methionine (M) did not abrogate the effect of T850D on AR activity (Figure 4 and Table S1). Methionine was selected as replacement residue instead of alanine because of the similar size of its side chain. While AR MRM and AR MMM demonstrate a reduced activity compared to AR WT, their activity is increased again by the T850D mutation (Table S1). This is reflected by a 33% increase of maximal activity of AR MRM-T850D compared to AR MRM (*p* < 0.05) and a 14% increase for AR MMM-T850D vs. AR MMM in COS-7 cells (*p* < 0.05). In PC3 cells, a 71% increase was found for AR MRM T850D vs. AR MRM (*p* < 0.05) and 76% increase for AR MMM-T850D vs. AR MMM (*p* < 0.05). In conclusion, the potentiating effect of T850D on AR activity is not dependent on electrostatic interactions with K845, R845 or K847.

### 2.5. AR T850D Gain of Function Due to Increased AF2 Interactions

**Figure 5:**
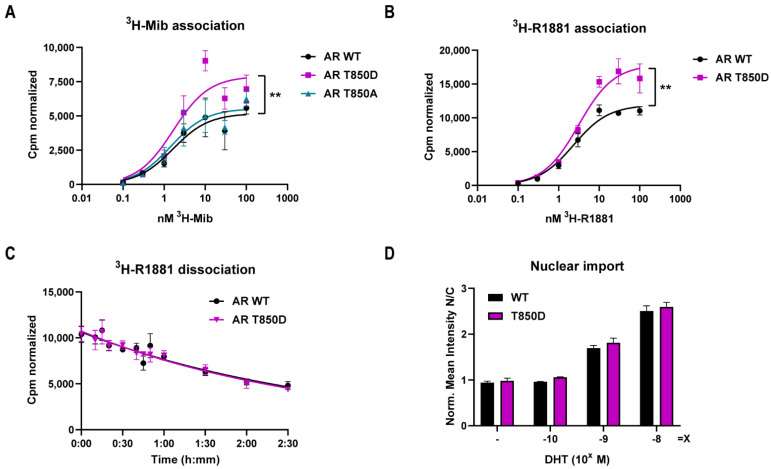
Effect of T850D on ligand binding and nuclear translocation of the AR. Binding of AR WT and AR T850D to ^3^H-Mib (**A**) and ^3^H-R1881 (**B**) in a whole-cell competition assay in COS-7 cells. Mean and SE of the normalized specific binding are depicted (*n* = 5 for ^3^H-Mib and *n* = 3 for ^3^H-R1881). K_D_ and B_max_ were calculated from the fitted curves after non-linear regression (Table 1). Ratio-paired t-test for specific binding curves with geometric means of ratios (B_max_T850D/B_max_WT) is 1.398 for ^3^H-Mib and 1.361 for ^3^H-R1881. B_max_ of AR WT vs. AR T850D is significantly different (** = *p* ≤ 0.01). (**C**) ^3^H-R1881 dissociation kinetics of AR WT and AR T850D. Mean values with SEM are given for each data point (*n* = 4). Dissociation half-lives were calculated after non-linear regression and given in Table 1. (**D**) Nuclear translocation of GFP-AR WT vs. GFP-AR T850D in Hela cells after 2 h of DHT incubation. Mean values with SEM are given for each data point (*n* ≥ 4).

Despite the fact that T850 is a surface residue on the LBD of AR, we decided to investigate the influence of T850D on ligand binding and concomitant nuclear translocation (Figure 5). In whole-cell competition experiments, AR T850D was found to bind ^3^H-Mib and ^3^H-R1881 with similar affinity (K_D_) as AR WT, although the maximal binding of both agonists was increased with approximately 50% (Figure 5A,B, Table 1). Ligand binding remained unaffected by the T850A mutation. Dissociation of ^3^H-R1881 from AR T850D was within the normal range, with a dissociation half-time of 121 min for AR T850D compared to 126 min for AR WT (Figure 5C, Table 1).

Using a GFP-tagged version of AR T850D, we quantified the potential to translocate to the nucleus. After 2 h of DHT stimulation, there is a concentration dependent increase of the nuclear/cytoplasmic ratio of both GFP-AR WT and GFP-AR T850D (Figure 5D). This ratio was similar for GFP-AR T850D vs. GFP-AR WT in the untreated and DHT-treated conditions.

**Figure 6:**
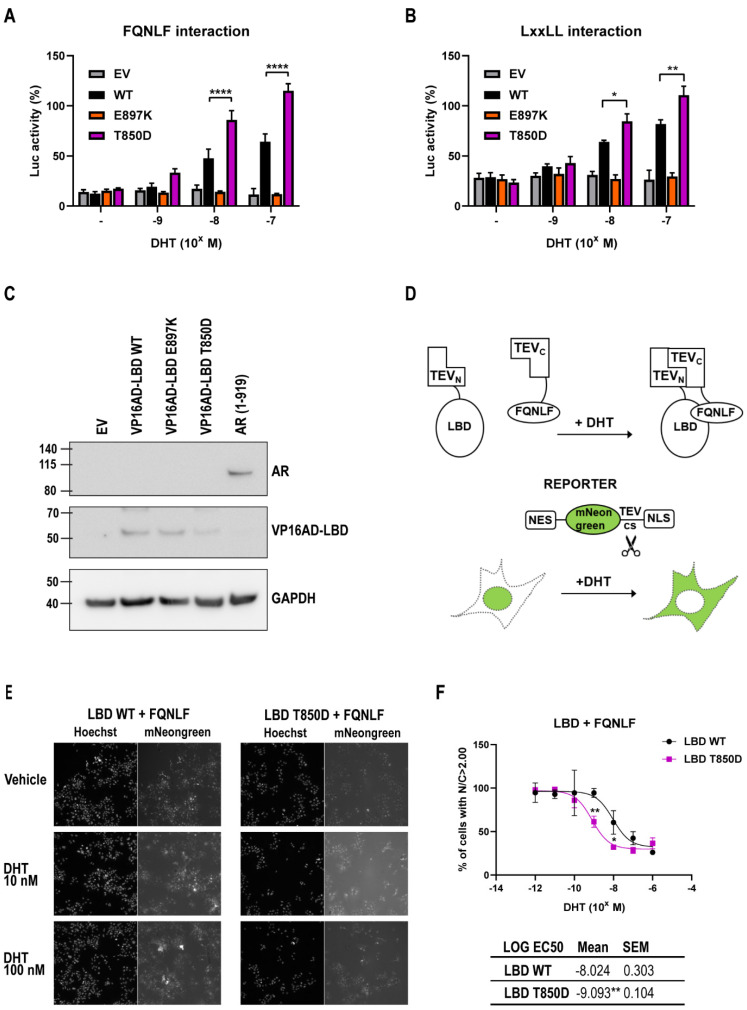
AF2 availability is affected by T850D. (**A**,**B**) Mammalian double hybrid assay to detect interaction between the AR LBD and the FQNLF or the LxxLL motif. The Gal4-DBD fused to AR 1–36 (**A**) or to SRC1a 1241–1441 (**B**) and VP16AD-LBD WT (black) or containing the indicated mutations E897K (orange), T850D (purple) or corresponding empty vector (grey) were co-transfected with the 5x Gal4 RE-Luc reporter. Mean and SEM are given for each data point (*n* = 4). Ordinary two-way ANOVA with the Tukey’s multiple comparisons test. * = *p* ≤ 0.05, ** = *p* ≤ 0.01 and **** = *p* < 0.001. (**C**) Western blot of wild type and mutated VP16AD-LBD. A DYKDDDDK Tag Antibody (Cell Signaling Technology) was used for detection of the Flag-tagged VP16 AD-LBD. Cells expressing a flag-tagged AR full-length were used as control. (**D**) Split TEV assay for N/C interaction. Interaction of TEV_N_-LBD with TEV_C_-FQNLF cleaves off the triple NLS from the mNeongreen reporter which results in the relocation of the reporter from nucleus to cytoplasm due to its remaining NES. (**E**) Cellular localization of the mNeongreen reporter in the stable Hep3B split TEV LBD WT and T850D cell line after 24 h-treatment with vehicle or DHT. (**F**) Dose-response curve showing the percentage of cells with predominantly nuclear staining (N/C > 2) in relation to DHT levels. LOG EC_50_ for DHT of the stable Hep3B split TEV LBD WT and T850D cell line are calculated. Unpaired, two tailed t-test was used to compare the LOG EC50, with * = *p* ≤ 0.05, ** = *p* ≤ 0.01.

The well-described interactions of the AF2-site of the AR LBD with the ^23^FQNLF^27^ motif in the AR NTD and the LxxLL-motifs present in co-regulators [36,37] were investigated by means of a mammalian double hybrid assay. Here, the FQNLF/LxxLL motif is fused to the Gal4-DBD and is allowed to interact with a VP16AD-LBD fusion protein. Interaction between LBD and the FQNLF/LxxLL motif initiates transcription of the GAL4-driven luciferase reporter. We observed that the AR LBD containing the T850D mutation initiates significantly more interaction with the ^23^FQNLF^27^ motif and with the LxxLL motif at the C-terminus of SRC1a (Figure 6A,B). The E897K mutation, which is located in the AF2 site, is known to disrupt both LxxLL and FQNLF interactions with the LBD [38]. Protein levels of LBD fusions were similar in all experiments (Figure 6C).

The effect of the T850D mutation on AF2 interactions with the ^23^FQNLF^27^ motif was further validated in a split TEV assay where we constructed fusion proteins containing either the amino-terminal region (TEV_N_) or the carboxyterminal part (TEV_C_) of the TEV protease. When TEV_N_-LBD interacts with TEV_C_-FQNLF, a functional TEV protease is reconstituted. A fluorescent mNeongreen reporter was designed to respond to TEV activity by translocation from the nucleus to the cytoplasm (Figure 6D and Figure S2). Both reporter and fusion proteins were stably integrated in Hep3B cells using the piggyBac system. At 10 nM and 100 nM, DHT induces the LBD-FQNLF interaction as indicated by the relocation of the split TEV reporter in both WT and T850D LBD (Figure 6E, Figure S3 and S4). The EC50 of the WT LBD for DHT (9.5 nM) is however 10-fold higher compared to the EC50 of the T850D LBD (0.81 nM) (Figure 6F). The LBD with T850D mutation thus demonstrates an increased interaction with the FQNLF motif.

### 2.6. Effects of T850D and T850 Phosphorylation on H10 and LBD Structure

**Figure 7:**
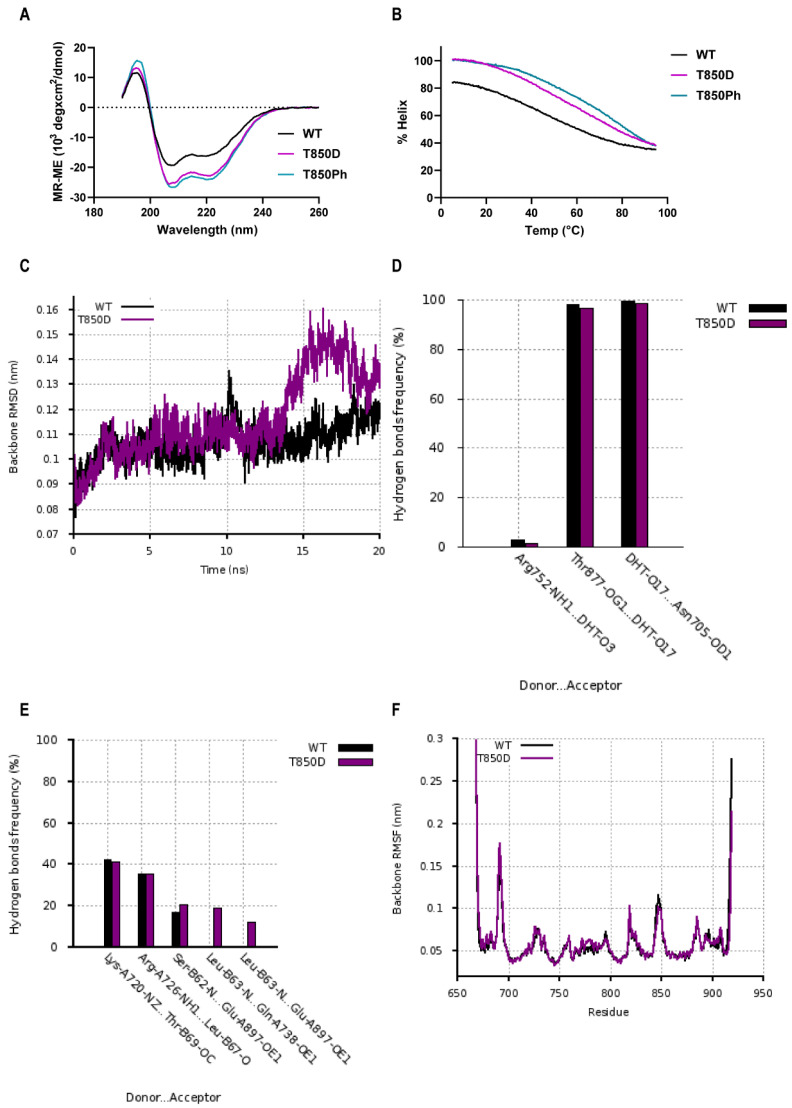
T850D and T850Ph modulate H10 and AF2. (**A**) CD spectra of H10-peptides (aa 845–868 of AR) with T850D or with phosphorylated T850 compared to WT peptide (50 µM peptide in 20% TFE-TRIS buffer pH 7.5). (**B**) Helical content of H10 peptides as a function of temperature. Helix content was calculated from the molar ellipticity [θ] at 222 nm (Figure S5) using the following equation, % helix = 100 ([θ]_222_/(−39,500(1 − 2.57/*n*))), where *n* is the number of total peptide bonds and [θ]_222_ has been corrected for background [40]. (**C**) Root-mean-square deviation (RMSD) of the LBD backbone for WT and T850D system. (**D**) Hydrogen bond frequency between residues of the AR LBP and DHT. (**E**) Hydrogen bond frequency between residues of the AF2 site within the AR LBD and the LxxLL peptide (^62^S**L**QF**LL**DT^68^ peptide) (**F**) Root-mean-square fluctuation (RMSF) of the backbone per residue of the AR LBD.

To elucidate how a residue at the N-terminus of H10 is able to influence the AF2-site on the other side of the LBD, we studied the effect of the phosphomimetic T850D and of T850 phosphorylation (T850Ph) on the structure of the isolated H10 and of the complete LBD. We investigated the helical properties of H10-containing peptides (aa 845–868) via Circular Dichroism (CD) (Figure 7). The minima at 208 nm and 222 nm demonstrate that the T850D/T850Ph peptide is more helical compared to the WT peptide (Figure 7A). While the T850D peptide shows a 30% and a 43% reduction (vs. WT) of the minima at 208nm and 222nm, respectively; phosphorylation of T850 even leads to an improved stabilization since the T850Ph peptide reduces the minima of the WT peptide with 38% and 51% at 208 nm and 222 nm, respectively. These findings confirm the predictions of increased helical content for the T850D modified peptide by AGADIR (Tables S2 and S3) [39]. Molar ellipticity at 222 nm was used to calculate helical content of each peptide sample in a temperature range from 5 °C to 95 °C with a 1 °C interval (Figure 7B). Thermal denaturation curves were determined at 4 different wave lengths (208 nm, 211 nm, 215 nm and 222 nm) (Figure S5). The melting temperature of the T850D peptide and the T850 phosphorylated peptide is approximately 13 °C and 27 °C higher than the WT peptide at all wave lengths, which shows that they have a more stable helical structure (Figure S5C). By stabilizing H10 which extends in H11, T850D/Ph might drive the LBD in a more active conformation.

To study the effect of the T850D mutation on the structure of the whole LBD, we performed Molecular Dynamic simulations. The structure of AR LBD monomer in complex with DHT and an AF2-binding peptide was extracted from the crystal structure homodimer (PDB: 5JJM) [10]. To access the stability of the WT and T850D simulated systems, the root-mean-square-deviation (RMSD) was calculated. Equilibration was reached in both systems after 2ns, exhibiting the same RMSD values of around 0.1 nm (1.0 Å) in the first 13ns-period (Figure 7C). After introduction of T850D, we observed similar hydrogen bond frequency with DHT in the LBP but additional hydrogen bond formation between AF2-residues and the ^62^SLQFLLDT^68^ co-activator peptide (Figure 7D,E). More specifically, the frequency of a hydrogen bond between L63 of the ^62^SLQFLLDT^68^ peptide and AR Q738 as well as AR E897 was increased for the T850D system. This finding was reproducible as it was observed in multiple simulations. While we observed the above-mentioned change in side chain interactions of AR and peptide, the backbone RMS fluctuations of the whole LBD protein remained minimal (Figure 7F). These MD simulations confirm the experimental findings that the T850D mutation did not affect ligand binding affinity but increased the availability of the AF2 site for protein-protein interactions.

## 3. Discussion

### 3.1. T850D Potentiates AR Independent from Reported Mechanisms

Threonine 850 is a known phosphorylation site in the human AR for which phosphorylation by PIM1L has been reported to stabilize the AR [27,35]. The higher transactivating potential of the phosphomimetic mutant AR T850D (Figure 1) and its advantage for proliferation (Figure 2) prompted us to investigate this gain-of-function. Despite its proximity to the dimerization interface centered around H5 [10], T850 phosphorylation is unlikely to regulate LBD dimerization because mutation of the positive patch (K845-R846-K847) positioned in the opposing LBD across the dimerization surface of T850D failed to abrogate the stimulatory effect of T850D on AR activity in our assay (Figure 4). In the light of the complexity of LBD dimerization by the oxosteroid receptors, the region of H9 and H10 has recently been suggested to participate in GRα and MR dimerization based on computational study of available LBD crystal structures [41]. Although there is no absolute conservation of the H9-H10 region within the oxosteroid receptors, T850D might play a role in alternative dimerization modes that are not yet experimentally validated for AR. Finally, the mutation of K845 and K847 to arginines only diminished the gain-of-function by T850D to some extent, ruling out ubiquitination by RNF6 as the only cause for AR stabilization after T850 phosphorylation (Figure 3) [27].

### 3.2. T850D Facilitates AF2 Binding by Allosteric Signaling

An allosteric pathway that is used by the heterodimeric nuclear receptors could explain the observed gain-of-function here. RXR is known to dimerize with permissive (PPARα, LXRα, etc.) and non-permissive partners (TRβ, VDR, etc.) leading to the potentiation or silencing of RXR activity in presence of both receptor ligands [42,43]. Extensive structural studies by Kojetin et al. pointed out that the presence of the ligand of the RXR-partner is transmitted through the H10-11 dimerization surface towards RXR [42]. Within RXR, the allosteric signal at the H10-11 interface leads to a rotation of H5 and ultimately affects the LBP and coactivator binding site (H3). The direction of the rotation of H5 indicates whether the binding partner of RXR and its ligand permit RXR to be active or inactive. Since H5 is at the core of the LBD and is part of the LBP and close to H3, the rotation transmits ligand occupancy of the RXR-partner into a change in ligand binding and coactivator peptide binding of RXR. While permissive heterodimeric partners stabilize RXR LBD in an active conformation, the non-permissive partners create instability at the LBP and AF2 site of RXR. This network seems evolutionary conserved for all heterodimeric nuclear receptors [44].

**Figure 8:**
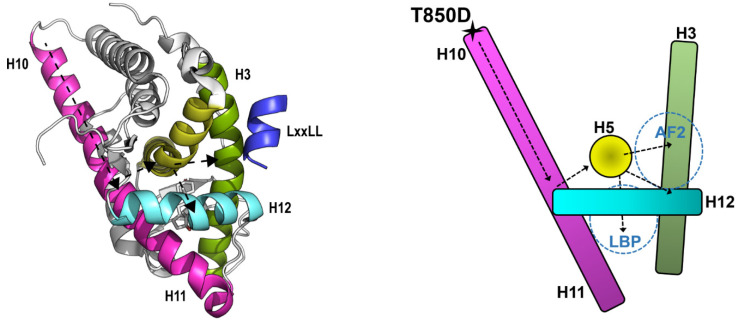
Allosteric pathway in AR LBD revealed by T850D. Proposed allosteric pathway starting at T850 and moving towards the AF2 site via H5. Numbering of the helices is according to the 12-helical canonical fold of NRs but helix 2 is lacking in AR LBD. We propose T850D to induce structural changes in H10-11 of AR that enable allosteric signaling towards AF2. Adapted from 5JJM using Pymol [34]. H3 (green), H5 (yellow), H10-11 (pink) and H12 (cyan) are indicated. The ^62^S**L**QF**LL**DT^68^ (LxxLL) peptide that binds in AF2 is depicted in blue. On the right, a simplified model is given.

Although AR does not use the H10-11 LBD surface for dimerization, the allosteric pathway starting at H10 and continuing towards H5 and H3 may still be functional (Figure 8). Surprisingly, some of the residues that are involved in the RXR pathway are indeed conserved in AR. P423 in H11 of RXR that converts the signal of LBD dimerization into rotation of H11 corresponds to P868 in AR. While W305 in H5 of RXR transmits the signal from H11 to H3 and to the LBP, it is W741 that could take over this role in AR. Besides this conservation, the following observations for T850D further confirm that this allosteric pathway is present in AR. (1) T850D/Ph induces a stabilization of H10 by increasing its helical content. (2) T850D leads to increased recruitment of coactivator peptides at AF2. (3) While the affinity and dissociation of ligands remains the same in AR T850D compared to AR WT, we observed a 50% increase of B_max_ in ligand binding experiments. The finding of an increased maximal amount of binding sites (B_max_) combined with equal protein expression suggests an increased percentage of AR in the active conformation. (4) MD simulations for T850D suggest the occurrence of an extended network of hydrogen bonds involved in the recruitment of the LxxLL coactivator motif. The presence of the allosteric network connecting H10-11 to H5 and H3 might explain why T850D located at the amino-terminus of H10 has an impact on the availability of the AF2 site on the other side of the LBD (Figure 8).

### 3.3. T850 Phosphorylation in PCa

While the T850D mutation has not been reported to occur in clinical samples in GenomAD [45], nor has it been reported in the McGill Androgen Receptor Gene Mutation Database [46] for occurring in PCa samples to date, T850 phosphorylation could be established by PIM1L or by other yet undefined kinases. Mechanistically, we observed that at least at the peptide level, phosphorylation leads to comparable structural adaptations in the LBD as the T850D phosphomimetic mutation. Moreover, the observed growth advantage at castrate levels of androgens of the lentiviral transduced LNCaP cells expressing T850D shows similarities with a study of Van Der Poel et al. where they observed a survival advantage for LNCaP cells stably transfected with PIM1 [47]. A recent study on circulating tumor cells in patients with metastatic castration resistant PCa reports that PIM1 is overexpressed in 37.5% of the cases [48]. Additionally, De Velasco et al. reported that PIM1 levels increased in 2 mouse models of Pten-deficient PCa when treating with apalutamide as monotherapy and an even further PIM1 increase when apalutamide was combined with an Akt inhibitor [49]. Due to increasing evidence that PIM1 overexpression is linked with PCa progression, it will be valuable to investigate the AR T850 phosphorylation status in clinical samples of primary and advanced PCa using phosphospecific antibodies as was done for S94, S308, S650 and S791 [50]. In this way, the prevalence of T850 phosphorylation and its importance in disease-progression can be estimated and the possibility of using T850 phosphorylation as a molecular marker can be explored.

## 4. Materials and Methods

### 4.1. Cells and Plasmids

PC3, COS-7 and Hela cells were obtained from ATCC (Manassas, VA, USA) and maintained in DMEM supplemented with 10% fetal calf serum, 1% Glutamax and 1% Penicillin-Streptomycin. The LNCaP cell line was obtained from ATCC (Manassas, VA, USA) and cultured as recommended. Hep3B cells were a kind gift of Prof. AB Houtsmuller (Erasmus MC, Rotterdam, The Netherlands) and maintained in EMEMα supplemented with 10% fetal calf serum, 1% Glutamax and 1% Penicillin-Streptomycin.

Two previously described plasmids [51], the pSG5-flag-wtAR plasmid and pEGFP-(GA)_6_-wtAR, were mutated using In-Fusion Technology (Clontech-Takara, San Jose, CA, USA). The reporter plasmid containing the pGL4-luciferase gene preceded by 4 repeats of the Slp-HRE2 (-4T-A) and the E1B TATA box was described before [52]. To correct for transfection efficiency, a pCMV-B-Galactosidase expression plasmid (Stratagene, La Jolla, CA, USA) is used. The plasmid containing the Gal4-DBD (1–147) fused to hAR FQNLF (1–36) was made by inserting a PCR-made fragment in the BamHI restriction site of pAB-Gal4 [53]. The plasmid containing the Gal4 DBD (1–147) fused to the LxxLL fragment of SRC1a (1241–1441) has been described before [54]. The (GAL4)_5_TATA-luc luciferase reporter plasmid was a kind gift of M.G. Parker (Imperial Cancer Research Fund, London, United Kingdom). To obtain the VP16 AD fused to AR LBD (640–919), a PCR-fragment containing the hAR LBD (640–919) was cloned in frame into the BglII site of pSNATCH-II [55]. Mutations (T850D or E897K) are inserted via In-Fusion Technology (Clontech-Takara, San Jose, CA, USA).

### 4.2. Transactivation Study

COS-7 or PC3 cells were seeded at a density of 10,000 cells per 96-well in DMEM, 5% stripped serum, 1% Glutamax and 1% Penicillin-Streptomycin. Ten ng of receptor plasmid, 100 ng of reporter plasmid and 5 ng of pCMV B-Gal plasmid were transfected in each well using GeneJuice Transfection Reagent (Novagen, Madison, WI, USA) or X-tremeGENE HP DNA Transfection Reagent (Roche, Basel, Switserland) for COS-7 and PC3 cells, respectively. The next day, cells were treated with increasing concentrations of DHT (from 0.1 nM to 1 µM). The following day, cells were lysed in Passive Lysis Buffer (Promega, Madison, WI, USA) and luciferase and B-Gal activity were measured using Luciferase Assay Reagent Buffer (Promega, Madison, WI, USA) or Galactolight Buffer, Galacton+ and Light Emission Accelerator (Applied Biosystems, Waltham, MA, USA). The relative luciferase activity for AR WT at 1 µM DHT was set at 100%, all other values are calculated accordingly. Fitted curves with mean and 95% CI area were calculated after non-linear regression using the log(agonist) vs. response formula with Hill slope 1 (three parameter) of GraphPad Prism 9, version 9.3.1, (*n* = 3–5). The Top (maximal activity) and EC50 were considered significantly different if 95% CI were not overlapping, * = *p* < 0.05.

### 4.3. Western Blot

Protein levels were determined in the cell lysates of the transactivation and double hybrid studies using the Coomassie (Bradford) Protein Assay Kit (Pierce). An equal amount of total protein supplemented with 4× LDS Sample Buffer and 10× Sample Reducing agent was loaded on a NuPAGE Bis-Tris 4–12% SDS-PAGE gel (ThermoFisher Scientific). After 1 h of semi-dry electrophoretic transfer onto a PVDF membrane, antibodies for AR (in-house antibody against NTD of AR, validation in Figure S6) and Flag-VP16AD-LBD (DYKDDDDK Tag antibody #2368 from Cell Signaling Technology, Danvers, MA, USA), GAPDH (MAB374 from EMD-Millipore, Burlington, MA, USA) or α-tubulin (T5168 from Sigma-Aldrich, St. Louis, MO, USA) were used for immunodetection. Visualization occurred after incubation with Western Lightning Plus-ECL (Perking Elmer, Waltham, MA, USA).

To asses androgen-induced stability of AR, PC3 cells were seeded at 50,000 cells per 24-well in DMEM, 5% stripped serum, 1% Glutamax and 1% Penicillin-Streptomycin. The next day, the cells were transfected with 250 ng AR WT or AR T850D expression plasmids. The next day, and after 2 h of stimulation with 10 nM DHT or vehicle, cells were harvested in Passive Lysis Buffer (Promega, Madison, WI, USA) and used for Western blot analysis according to the protocol mentioned above.

### 4.4. Generation of Lentiviral LNCaP Cell Lines

For AR replacement experiments, the pCS-CG plasmid was adapted to contain a U6-driven shRNA against AR and to contain a CMV-flag-AR-P2A-T2A-GFP cassette with codon switched AR at residues A811-S816. Plasmid pCS-CG was a gift from Inder Verma (Addgene plasmid # 12154) [56]. The sequence of the AR shRNA is (AR target sequence underlined) 5′-GCACTGCTACTCTTCAGCATTCAAGAGATGCTGAAGAGTAGCAGTGCTTTTTT-3′, adapted from Guo et al. [57]. To introduce mutations in the AR (T850A and T850D), a PCR-fragment encoding the mutated and codon switched AR LBD is inserted in the XbaI-Tth111I sites of the final plasmid (pCS-CG shAR + AR WT codon switched).

Viral particles were collected 48h after transfection of HEK 293T cells with 450ng psPAX2, 50ng pVSV.G and 500ng of the pCS-CG lentiviral plasmid with cassette of interest in a 6-well. psPAX2 was a gift from Didier Trono (Addgene plasmid # 12260). VSV.G was a gift from Tannishtha Reya (Addgene plasmid # 14888) [58]. Polybrene (Millipore, Burlington, MA, USA) was added to a final concentration of 4 µg/mL and the medium was transferred to wells containing LNCaP cells at 70% confluency. The next day, viral particles were removed and LNCaP cells were further cultured as recommended by ATCC (Manassas, VA, USA).

### 4.5. Proliferation Measurements

The proliferation measurements were performed by live cell analysis (IncuCyte ZOOM, Essen BioScience, Newark, United Kingdom). Transduced LNCaP cells were seeded in Poly-L-Lysin coated, black 96-well plates with transparent bottoms at a density of 4000 cells/well. R1881 was diluted in medium supplemented with CSS (charcoal-stripped serum) and immediately added to the wells. The starting concentration of R1881 was 1nM and this was further serially diluted with a dilution factor of 3, covering a range between 1.4pM to 1nM R1881. Green Object Confluence (percent) was recorded over time. The proliferation rate (slope of growth curve) was calculated once the cells maintained a stable growth rate.

### 4.6. Ligand Binding and Dissociation

Ligand binding properties of AR WT and AR T850D were investigated by a whole-cell competition assay in COS-7 cells with ^3^H-Mib and ^3^H-R1881. COS-7 cells were seeded at a density of 30,000 cells per 48-well in DMEM, 5% stripped serum, 1% Glutamax and 1% Penicillin-Streptomycin. The next day, 375 ng of expression plasmid for AR WT and AR T850D was transiently transfected using GeneJuice Transfection Reagent (Novagen, Madison, WI, USA). Two days later, binding of the receptor to a series of ^3^H-R1881 concentrations (0.1 nM; 0.3 nM; 1 nM: 3 nM; 10 nM; 30 nM and 100 nM) was tested, both in absence (total binding) and in presence of a >100-fold molar excess of DHT (non-specific binding). After 90 min of incubation at 37 °C, cells were washed with ice-cold PBS three times and lysed using Passive Lysis Buffer (Promega, Madison, WI, USA). After addition of Ultima Gold XR (Perkin Elmer, Waltham, MA, USA), cell lysates were counted for ^3^H-signal. Data were normalized for the average cpm of all data points per independent experiment. Mean and SEM of the specific binding (total binding—non-specific binding) is given (*n* ≥ 3). Via non-linear regression analysis using the One-site binding-equation of GraphPad Prism 9 (version 9.3.1), the K_D_ and B_max_ were calculated. Ratio-paired T-test with * = *p* ≤ 0.05, ** = *p* < 0.01.

The dissociation rate of ^3^H-R1881 from the receptor was studied in whole-cell binding assays. Similar to the ligand binding assay, COS-7 cells were transfected with 375 ng receptor plasmid per 48-well. After 2 h of incubation with 5 nM ^3^H-R1881, a 10,000-fold excess of R1881 was added to the wells to initiate ligand dissociation. Cells were lysed at different time points (up to 2.5 h) to determine the remaining ^3^H-R1881 binding via scintillation counting. Dissociation half-time is calculated via non-linear regression analysis using the equation ‘Dissociation—One phase exponential decay’ of GraphPad Prism 9 (version 9.3.1). Mean and SEM of 4 independent experiments are given.

### 4.7. Nuclear Translocation

Nuclear translocation of GFP-AR WT vs. GFP-AR T850D was followed up with the CellInsight CX5 (ThermoFisher Scientific, Waltham, MA, USA). Ten ng of GFP-fused receptor plasmid and 80 ng empty vector were transfected per 96-well of Hela cells. The next day, DHT was added at 0.1, 1 and 10 nM for a 2-h incubation. Cells were fixated and nuclei were stained with Hoechst33342 (Sigma-Aldrich, St. Louis, MO, USA). The average intensity of the GFP signal was calculated for nucleus and cytoplasm of each transfected Hela cell (>50 cells/96-well and the ratio of average intensity in nucleus/cytoplasm is given as the mean ratio with SEM (*n* = 4). Multiple paired T-test, correction for Multiple comparisons via the Holm-Sidak method, * = *p* ≤ 0.05.

### 4.8. Mammalian Double Hybrid for LBD Interaction with FQNLF or LXXLL

To study interaction between the AR LBD and the FQNLF motif of AR or the LxxLL motif of SRC1a, COS-7 cells were seeded at a density of 10,000 cells er 96-well in DMEM, 5% stripped serum, 1% Glutamax and 1% Penicillin-Streptomycin. The next day, 100 ng (Gal4)5 TATA Luc reporter plasmid, 50 ng of plasmid encoding Gal4 DBD—AR (1–36) or Gal4 DBD—SRC1a (1241–1441), 10 ng of plasmid expressing VP16 AD—AR LBD (640–919) and 5 ng of pCMV-B-Gal are co-transfected per 96-well. For stimulation and harvesting of the cells, the protocol for the transactivation study was followed. Data was normalized for the average Luc activity of all data points per independent experiment (set at 100%). Mean and SEM of 4 independent experiments are depicted. Ordinary two-way ANOVA and the Tukey’s multiple comparisons test with * = *p* ≤ 0.05, ** = *p* ≤ 0.01 and **** = *p* < 0.0001.

### 4.9. Split-TEV Assay for LBD-FQNLF Interaction

A piggyBac^TM^ transposon plasmid was constructed for stable integration in Hep3B cells. Within the transposon, two reading frames in opposite directions were constructed starting from the EF1 promoter or the mPGK promoter. The EF1 promotor drives expression of 3xflag-TEV_N_-hLBD (662–919)-SV40 NLS and SV40 NLS-3xHA-TEV_C_-(Gly-Ala)_6_-FQNLF separated by the tandem ribosome skipping site (P2A-T2A). The mPGK promotor drives the split TEV reporter (PKI NES-mNeongreen-mNeongreen-3x nucleoplasmin NLS) and the puromycin resistance cassette separated by IRES2. Hep3B cells were transfected using Fugene6 (Promega, Madison, WI, USA) with a 5:1 ratio of the piggyBac^TM^ transposon plasmid and the piggyBac^TM^ transposase plasmid. After 48 h, Puromycin was added at 0.5 µg/µL and concentration increased gradually over time to max 1.5 µg/µL. After 2 weeks, cells with stable integration were identified in the Incucyte Zoom (Essen BioScience, Newark, UK). Cell lines were maintained in 0.75 µg/mL Puromycin final concentration.

To determine LBD-FQNLF interaction, 5000 cells were seeded in a black, Poly-L-lysin coated 96-well with transparent bottom in EMEMα with 5% stripped-FCS, 1% Glutamax and 1% Penicillin-Streptomycin. The next day, DHT was added in different concentrations (1 pM to 1 µM) and incubated overnight. Cells were subsequently fixed in 10% formalin, permeabilized in 0.2% Triton X-100 in PBS and stained with Hoechst33342 in PBS (2 µg/mL). Nuclear and cytoplasmic mNeongreen signal in the cells was quantified using the CellInsight CX5 (ThermoFisher Scientific, Waltham, MA, USA) of the laboratory of Prof D. Daelemans, Rega Institute, KU Leuven. Ten fields were taken from each 96-well and, for each field, cells were selected based on their simultaneous Hoechst and mNeongreen staining. At least 500 cells/96-well were taken for analysis. Nuclei were identified by the Hoechst staining of the DNA. A Circle was defined 2 pixels inwards from the actual nucleus border. A Ring corresponding to the cytoplasmic region was defined from 2–6 pixels outwards from the nuclear border. The mNeongreen intensity was determined for both regions (Circle and Ring) in each selected cell and was divided by the area to obtain Average Intensities of Circle and Ring. The average of the Circle to Ring ratio is calculated for each cell. Via non-linear regression using the ‘log(agonist) vs. response’ formula with Hill slope -1 (three parameter) the LOG EC50s are calculated. Unpaired, two tailed t-test was used to compare the LOG EC50, with ** = *p* ≤ 0,01. Mean and SEM of >4 independent experiments are given. Ordinary two-way ANOVA with the Tukey’s multiple comparisons test was applied on the DHT-curve, * = *p* ≤ 0.05, ** = *p* ≤ 0.01.

### 4.10. Circular Dichroism Spectroscopy

Peptides (AR WT 845–868: Acetyl-KRKNP**T**SCSRRFYQLTKLLDSVQP-amide) with T850D or with phosphorylated T850 (Genscript, Piscataway, NJ, USA) were compared to WT peptide through their CD spectra. The CD spectra at 4 °C were measured in 20% TFE—50 mM TRIS buffer pH 7.5 at a peptide concentration of 50 µM using a quartz cuvette with 0.1 cm pathlength and with a Jasco J-1500 spectropolarimeter. The Mean Residue-Molar Ellipticity (MR-ME) was calculated as MR-ME = MRW × θ/(10 × d × c) with MRW (Mean Residue Weight) = peptide weight/(*n −* 1) and *n* = amount of peptides; with θ as the observed ellipticity in millidegrees; with d as the pathlength in cm and with c as the concentration in g/L. Four wave-lengths (208 nm, 211 nm, 215 nm and 222 nm) were selected for thermal denaturation of the peptides (5–95 °C with 0.2 °C interval). For thermal denaturation, peptides were diluted to 25 µM in 20% TFE—50 mM TRIS buffer pH 7.5 and measured using a cuvette with 0.2 cm pathlength. The unfolded fraction (F_unfolded_) of the peptide was calculated from the Ellipticity data using equation: F_unfolded_ = (θ_t_ − θ_folded_)/(θ_unfolded_ − θ_folded_) with θ_t_ as the Ellipticity at each temperature, θ_folded_ as the Elllipticity of the peptide in folded state and θ_unfolded_ as the Ellipticity of the peptide in unfolded state. Melting temperature (T_m_) of AR 845–868 peptides were calculated through nonlinear regression analysis of the Sigmoidal dose response curves (variable slope) using GraphPad Prism 9, version 9.3.1. The temperature corresponding to an unfolded fraction of 0.5 is the T_m_. Mean melting temperatures with 95% CI of 2 independent experiments are given. Helix content was calculated from the molar ellipticity [θ] at 222 nm using the following equation, % helix = 100 ([θ]_222_/(−39,500(1 − 2.57/*n*))), where *n* is the number of total peptide bonds and [θ]_222_ has been corrected for background [40].

### 4.11. MD Simulations

To investigate the effect of T850D on the interaction between the AR LBD monomer and the AF2 peptide, we relied on molecular dynamics (MD) simulations. The structure of the AF2 peptide bound AR LBD; consisting of the AR LBD monomer, DHT and AF2 peptide; was extracted from the AR LBD crystal structure homodimer (PDB: 5jjm) [10]. The T850D mutation was introduced to the system. The wildtype and mutated structures were protonated and minimized by means of the MOE Protonate3D and Energy Refinement functions [59]. ACPYPE (AnteChamber PYthon Parser interfacE) was used to generate the topology for DHT. The Gromacs v.2019.3 package was used to perform the MD simulations [60]. In the simulations, the amber99sb force field [61] was applied to generate the topology for the protein and peptide. The complex systems were placed in a rhombic dodecahedron solvent box with the periodic distance of 0.85 nm using the three-points (TIP3P) water model [62]. In order to neutralize the systems’ charge, 6, 4 Cl^−^ ions were added to the solvent boxes of the WT and mutant, respectively. Next, steepest descent-based energy minimization was carried out, followed by systems equilibrations including 100 ps V-rescale thermostat NVT ensemble, 100 ps V-rescale thermostat and Parrinello-Rahman barostat NPT ensemble [63,64]. Finally, the MD production steps were performed for 20 ns by using the High-Performance Computer (HPC) of Vlaams Supercomputer Center, Belgium. The simulations were run in triplicate. During the equilibration and the production, all bond lengths were constrained using the LINCS algorithm [65]. The long-range electrostatic interactions were treated via PME implementation of the Ewald method with a cut-off value of 1.1 nm and the van der Waals interactions were modeled with a 1.4 nm cut-off [66].

## 5. Conclusions

Functional and structural studies pointed out that the AR T850D mutation is an exceptional gain-of-function mutation on the surface of the LBD in H10. While the T850D mutation does not affect ligand binding affinity, it does enhance co-regulator binding at AF2 by promoting the active conformation of the AR LBD. There is resemblance with an allosteric network in the heterodimeric NRs where the H10-11 dimerization interface transmits permissive or non-permissive signals from one LBD to the other. Via rotation of H5, signals at H10-11 are passed on to the LBP and to AF2. Modulation of this allosteric pathway could provide a new mechanistic explanation for the presence of AR-related diseases such as PCa and the androgen insensitivity syndrome. These findings provide new details on the allosteric pathways within the LBD of AR [67].

## Figures and Tables

**Table 1:** Equilibrium binding and dissociation of ^3^H-Mib and ^3^H-R1881. Binding affinity (K_D_ and B_max_) and dissociation half-times (t_1/2_) are given for AR WT, AR T850A and AR T850D. Mean ± SE are given. ** = *p* < 0.01.

	^3^H-Mib Binding		^3^H-R1881 Binding		^3^H-R1881 Dissociation
	K_D_ (nM)	B_max_ (cpm)	K_D_ (nM)	B_max_ (cpm)	t_1/2_ (min)
**AR WT**	1.65 (±0.92)	5182 (±602)	2.32 (±0.43)	11,905 (±488)	126.4 (±21.4)
**AR T850A**	1.45 (±0.72)	5514 (±561)			
**AR T850D**	1.67 (±0.74)	7902 (±773) **	3.11 (±0.72)	17,849 (±959) **	120.8 (±21.7)

## Data Availability

Data is contained within the article or Supplementary Materials.

## Supplementary Materials

The following are available online at https://www.mdpi.com/article/10.3390/ijms23031557/s1
