# Supplementary material for: The T850D Phosphomimetic Mutation in the Androgen Receptor Ligand Binding Domain Enhances Recruitment at Activation Function 2
Source: Int J Mol Sci. 2022 Jan 29;23(3):1557. doi: 10.3390/ijms23031557 (PMC8836279; doi:10.3390/ijms23031557)
Supplement: Supplementary file 1 [file ijms-23-01557-s001.zip › ijms-1528030-supplementary.pdf]

The T850D phosphomimetic mutation in the androgen receptor ligand binding domain enhances recruitment at AF2.

Christine Helsen[1*], Tien Nguyen[2], Thomas Vercruysse[3], Staf Wouters[2], Dirk Daelemans[3], Arnout Voet[2], Frank Claessens[1]

Table S1: EC50, maximal activity (Top) and p-values of AR mutants.

Table S2: Helical and N-capping contribution of H10 peptide residues according to AGADIR.

Table S3: Output data of AGADIR for H10 peptide: WT, T850D and T850A.

Figure S1: Expression levels of mutant receptors in COS-7 and PC3 cells.

Figure S2: Split TEV assay validation.

Figure S3: Magnification of split TEV images for LBD WT and FQNLF interaction.

Figure S4: Magnification of split TEV images for LBD T850D and FQNLF interaction.

Figure S5: Thermal denaturation curves of H10 peptides at 208 nm, 211 nm, 215 nm and 222 nm.

Figure S6: Validation of in-house AR antibody.

Figure S7: Validation of shAR for knock-down in LNCaP.

Table S1: EC50, maximal activity (Top) and p-values of AR mutants.

	COS-7		PC-3	
	EC50 (nM)	Top (%)	EC50 (nM)	Top (%)
**AR WT**	4.89	97.9	0.20	99.0
**AR T850A**[a]	4.66	102.0	0.46	119.4
**AR T850D**[a]	1.47*	158.2*	0.44	231.2*
**AR K845R**[a]	5.31	90,4	0.31	105.8
**AR K847R**[a]	5.06	83.7	0.66	107.3

[a] Mutants are compared to AR WT.

	COS-7		PC-3	
	EC50 (nM)	Top (%)	EC50 (nM)	Top (%)
**AR RRR**	30,9	86.0	0.21	87.7
**AR RRR-T850D**[b]	11,5	110.0*	0.15	181.9*
**AR MRM**	247,3	54.9	0.85	90.4
**AR MRM-T850D**[b]	69.2*	88.5*	0.32	161.8*
**AR MMM**	220.9	27.4	0.79	48.8
**AR MMM-T850D**[b]	82.7*	41,3*	0.37	124.3*

[b] T850D mutants are compared to corresponding mutant AR, for example RRR-T850D vs RRR.

Table S2: Helical and N-capping contribution of H10 peptide residues according to AGADIR.

Agadir		Helical contribution			N-capping contribution		
res	aa	T850	D850	A850	T850	D850	A850
845	K	0.0,	0.0,	0.0,	0.00,	0.00,	0.00,
846	R	0.0,	0.0,	0.0,	0.00,	0.00,	0.00,
847	K	0.0,	0.0,	0.0,	0.00,	0.00,	0.00,
848	N	0.0,	0.0,	0.0,	0.15,	0.40,	0.41,
849	P	0.2,	0.4,	0.4,	0.04,	0.17,	0.10,
850	x	0.2,	0.6,	0.5,	1.22,	1.02,	0.17,
851	S	1.4,	16.1,	0.7,	3.84,	13.85,	3.87,
852	C	5.3,	15.4,	4.5,	0.56,	0.59,	0.56,
853	S	5.8,	16.0,	5.0,	1.80,	1.61,	1.82,
854	R	7.6,	17.6,	6.8,	0.20,	0.18,	0.20,
855	R	7.6,	17.6,	6.9,	0.25,	0.22,	0.25,
856	F	7.4,	16.5,	6.7,	0.18,	0.16,	0.18,
857	Y	6.9,	14.8,	6.3,	0.44,	0.39,	0.45,
858	Q	6.9,	14.3,	6.4,	0.53,	0.47,	0.54,
859	L	7.2,	14.2,	6.7,	0.08,	0.07,	0.08,
860	T	7.2,	14.3,	6.7,	0.00,	0.00,	0.00,
861	K	6.8,	13.4,	6.4,	0.00,	0.00,	0.00,
862	L	6.3,	12.3,	5.9,	0.00,	0.00,	0.00,
863	L	5.8,	11.3,	5.4,	0.00,	0.00,	0.00,
% Helix of 845-863		4,34	9,49	3,96			

Table S3: Output data of AGADIR for H10 peptide: WT, T850D and T850A.

```
http://agadir.crg.es
Copyright @1997-2002, Lacroix E., Munoz V., Petukhov M. & Serrano, L.

pH                  7
Temperature         278
Ionic Strength      0.1

Nterm               acetylated
Cterm               amidated

Peptide 1    KRKNPTSCSRRFYQLTKLL
res,  aa,  Hel,  Ncap,  Ccap,Hstaple,Schellm, CaH,  13Ca, JaN
0.000010
00,   B,   0.0,  0.00,  0.00,  0.00,  0.00,  0.00 ,  0.00,  0.00
01,   K,   0.0,  0.00,  0.00,  0.00,  0.00, -0.00 ,  0.00,  6.41
02,   R,   0.0,  0.00,  0.00,  0.00,  0.00, -0.00 ,  0.00,  6.46
03,   K,   0.0,  0.00,  0.00,  0.00,  0.00, -0.00 ,  0.00,  6.41
04,   N,   0.0,  0.15,  0.00,  0.00,  0.00,  0.28 , -2.20,  7.04
05,   P,   0.2,  0.04,  0.00,  0.00,  0.00, -0.00 ,  0.00,  6.00
06,   T,   0.2,  1.22,  0.00,  0.00,  0.00, -0.00 , -0.02,  7.47
07,   S,   1.4,  3.84,  0.00,  0.00,  0.00, -0.00 , -0.04,  6.66
08,   C,   5.3,  0.56,  0.00,  0.00,  0.00, -0.02 ,  0.07,  7.02
09,   S,   5.8,  1.80,  0.02,  0.00,  0.00, -0.01 ,  0.12,  6.55
10,   R,   7.6,  0.20,  0.04,  0.00,  0.00, -0.06 ,  0.23,  6.36
11,   R,   7.6,  0.25,  0.16,  0.00,  0.00, -0.12 ,  0.23,  6.38
12,   F,   7.4,  0.18,  0.45,  0.00,  0.00, -0.09 ,  0.24,  6.97
13,   Y,   6.9,  0.44,  0.72,  0.00,  0.00, -0.11 ,  0.21,  7.12
14,   Q,   6.9,  0.53,  0.41,  0.00,  0.00, -0.15 ,  0.19,  6.35
15,   L,   7.2,  0.08,  0.25,  0.00,  0.00, -0.06 ,  0.13,  6.48
16,   T,   7.2,  0.00,  0.01,  0.00,  0.00, -0.04 ,  0.25,  7.29
17,   K,   6.8,  0.00,  0.42,  0.00,  0.00, -0.03 ,  0.17,  6.35
18,   L,   6.3,  0.00,  0.53,  0.00,  0.00, -0.02 ,  0.12,  6.52
19,   L,   5.8,  0.00,  0.51,  0.00,  0.00, -0.02 ,  0.11,  6.70
20,   U,   0.0,  0.00,  5.77,  0.00,  0.00,  0.00 ,  0.00,  0.00
Percentage helix         4.34

Peptide 2    KRKNPDSCSRRFYQLTKLL
res,  aa,  Hel,  Ncap,  Ccap,Hstaple,Schellm, CaH,  13Ca, JaN
0.000042
00,   B,   0.0,  0.00,  0.00,  0.00,  0.00,  0.00 ,  0.00,  0.00
01,   K,   0.0,  0.00,  0.00,  0.00,  0.00, -0.00 ,  0.00,  6.41
02,   R,   0.0,  0.00,  0.00,  0.00,  0.00, -0.00 ,  0.00,  6.46
03,   K,   0.0,  0.00,  0.00,  0.00,  0.00, -0.00 ,  0.00,  6.41
04,   N,   0.0,  0.40,  0.00,  0.00,  0.00,  0.28 , -2.20,  7.05
05,   P,   0.4,  0.17,  0.00,  0.00,  0.00, -0.00 ,  0.01,  6.01
06,   D,   0.6,  1.02,  0.00,  0.00,  0.00,  0.01 , -0.09,  6.61
07,   S,   1.6, 13.85,  0.00,  0.00,  0.00,  0.00 , -0.23,  6.84
08,   C,  15.4,  0.59,  0.00,  0.00,  0.00, -0.07 ,  0.20,  6.80
09,   S,  16.0,  1.61,  0.02,  0.00,  0.00, -0.04 ,  0.40,  6.37
10,   R,  17.6,  0.18,  0.03,  0.00,  0.00, -0.08 ,  0.53,  6.20
11,   R,  17.6,  0.22,  0.18,  0.00,  0.00, -0.13 ,  0.53,  6.25
12,   F,  16.5,  0.16,  1.25,  0.00,  0.00, -0.13 ,  0.55,  6.86
13,   Y,  14.8,  0.39,  1.92,  0.00,  0.00, -0.13 ,  0.46,  7.03
14,   Q,  14.3,  0.47,  0.88,  0.00,  0.00, -0.15 ,  0.39,  6.28
15,   L,  14.2,  0.07,  0.52,  0.00,  0.00, -0.08 ,  0.27,  6.38
16,   T,  14.3,  0.00,  0.03,  0.00,  0.00, -0.08 ,  0.50,  7.12
17,   K,  13.4,  0.00,  0.88,  0.00,  0.00, -0.06 ,  0.33,  6.30
18,   L,  12.3,  0.00,  1.09,  0.00,  0.00, -0.05 ,  0.24,  6.48
19,   L,  11.3,  0.00,  1.04,  0.00,  0.00, -0.04 ,  0.22,  6.81
20,   U,   0.0,  0.00, 11.27,  0.00,  0.00,  0.00 ,  0.00,  0.00
Percentage helix         9.49

Peptide 3    KRKNPASCSRRFYQLTKLL
res,  aa,  Hel,  Ncap,  Ccap,Hstaple,Schellm, CaH,  13Ca, JaN
0.000049
00,   B,   0.0,  0.00,  0.00,  0.00,  0.00,  0.00 ,  0.00,  0.00
01,   K,   0.0,  0.00,  0.00,  0.00,  0.00, -0.00 ,  0.00,  6.41
02,   R,   0.0,  0.00,  0.00,  0.00,  0.00, -0.00 ,  0.00,  6.46
03,   K,   0.0,  0.00,  0.00,  0.00,  0.00, -0.00 ,  0.00,  6.41
04,   N,   0.0,  0.41,  0.00,  0.00,  0.00,  0.28 , -2.20,  7.05
05,   P,   0.4,  0.10,  0.00,  0.00,  0.00, -0.00 ,  0.01,  6.00
06,   A,   0.5,  0.17,  0.00,  0.00,  0.00, -0.00 ,  0.01,  5.68
07,   S,   0.7,  3.87,  0.00,  0.00,  0.00,  0.00 , -0.06,  6.67
08,   C,   4.5,  0.56,  0.00,  0.00,  0.00, -0.02 ,  0.06,  7.04
09,   S,   5.0,  1.82,  0.07,  0.00,  0.00, -0.01 ,  0.10,  6.57
10,   R,   6.8,  0.20,  0.09,  0.00,  0.00, -0.06 ,  0.20,  6.37
11,   R,   6.9,  0.25,  0.09,  0.00,  0.00, -0.12 ,  0.21,  6.38
12,   F,   6.7,  0.18,  0.38,  0.00,  0.00, -0.09 ,  0.22,  6.97
13,   Y,   6.3,  0.45,  0.62,  0.00,  0.00, -0.11 ,  0.19,  7.12
14,   Q,   6.4,  0.54,  0.37,  0.00,  0.00, -0.15 ,  0.18,  6.36
15,   L,   6.7,  0.08,  0.23,  0.00,  0.00, -0.06 ,  0.12,  6.49
16,   T,   6.7,  0.00,  0.01,  0.00,  0.00, -0.04 ,  0.24,  7.30
17,   K,   6.4,  0.00,  0.39,  0.00,  0.00, -0.03 ,  0.16,  6.35
18,   L,   5.9,  0.00,  0.49,  0.00,  0.00, -0.02 ,  0.11,  6.53
19,   L,   5.4,  0.00,  0.48,  0.00,  0.00, -0.02 ,  0.11,  6.69
20,   U,   0.0,  0.00,  5.39,  0.00,  0.00,  0.00 ,  0.00,  0.00
Percentage helix         3.96
```

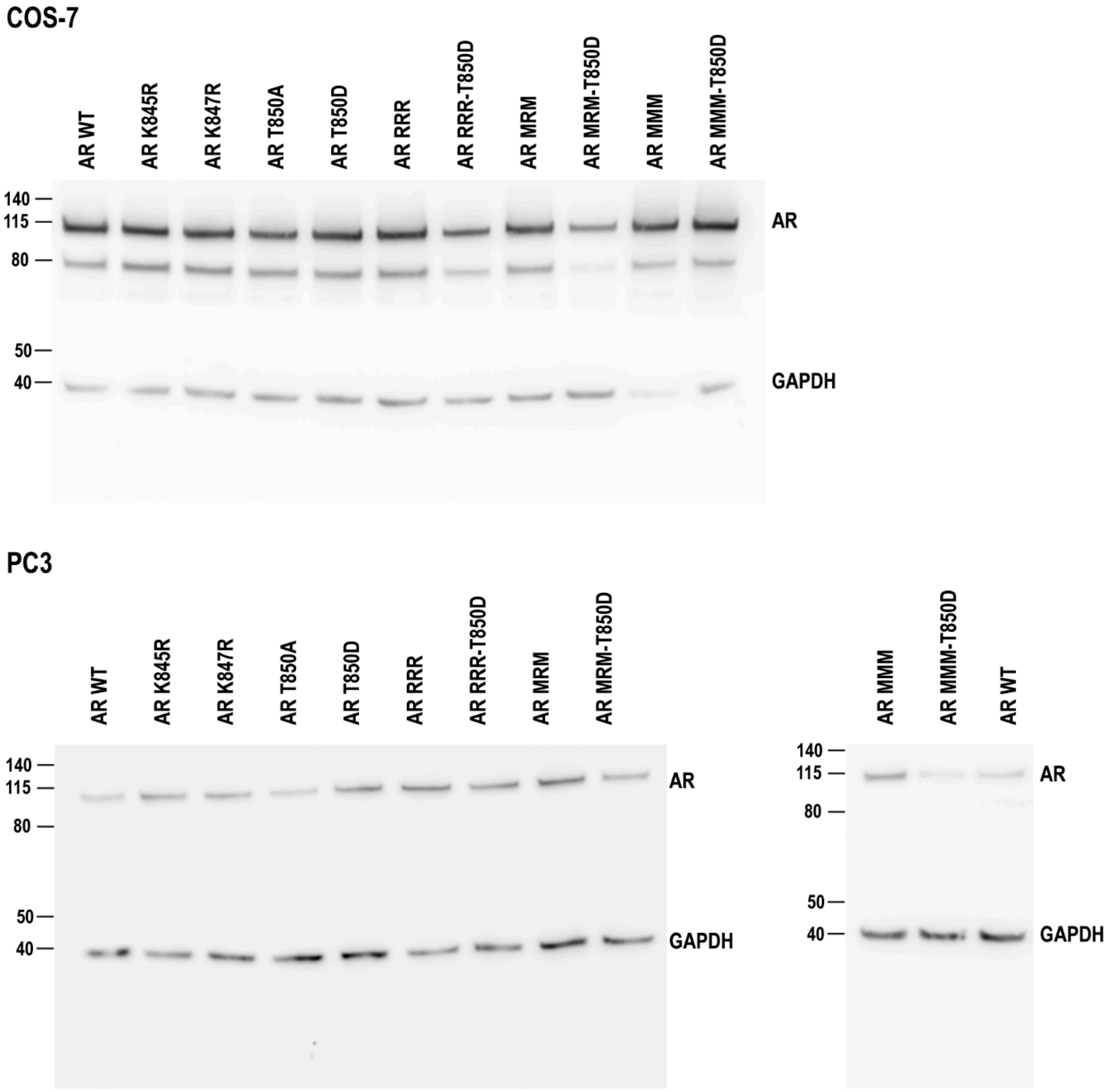

Figure S1: Expression levels of mutant receptors in COS-7 and PC3 cells. AR levels have been determined in the cell extracts of transfections in Figure 1, 2 and 3. GAPDH is used as household reference protein. Representative Western blots are given.

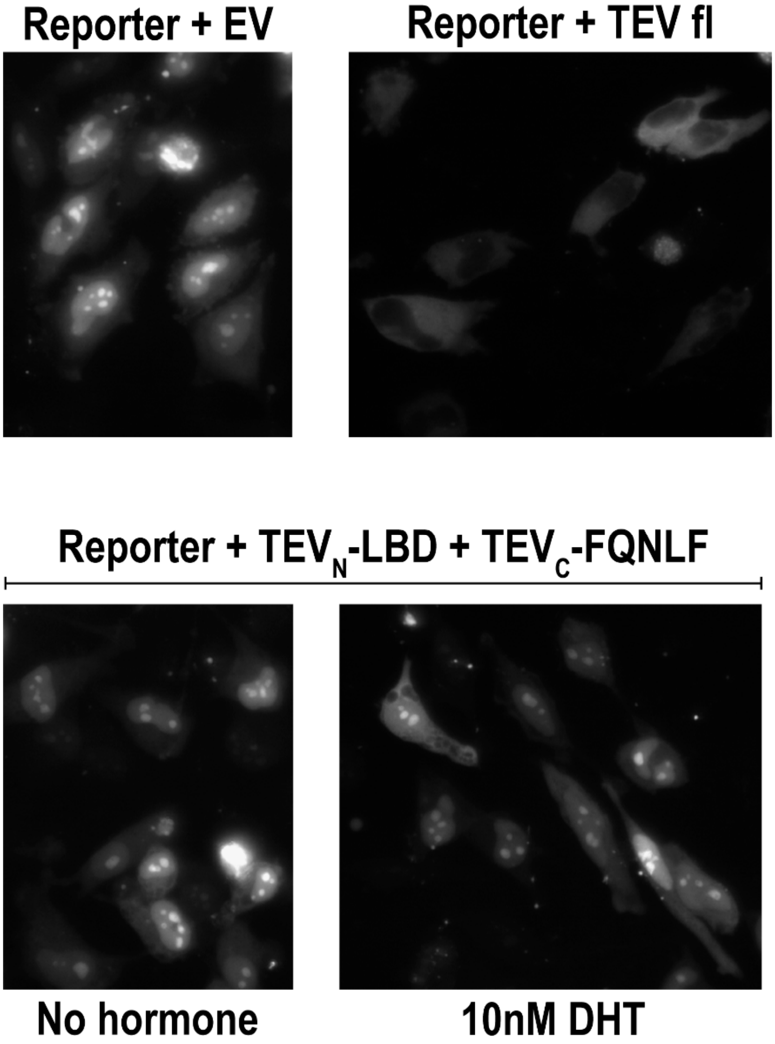

Figure S2: Split TEV assay validation. The localization of the split TEV mNeongreen reporter was monitored after transient co-transfection of the empty vector (EV), the expression plasmid for the full-length TEV protease (TEV fl) or both fusion proteins: TEV$_N$-LBD and TEV$_C$-FQNLF. The TEV protease is able to cause a complete shift of the split TEV reporter from nucleus to cytoplasm, while the fusion proteins increase the cytoplasmic presence of the reporter.

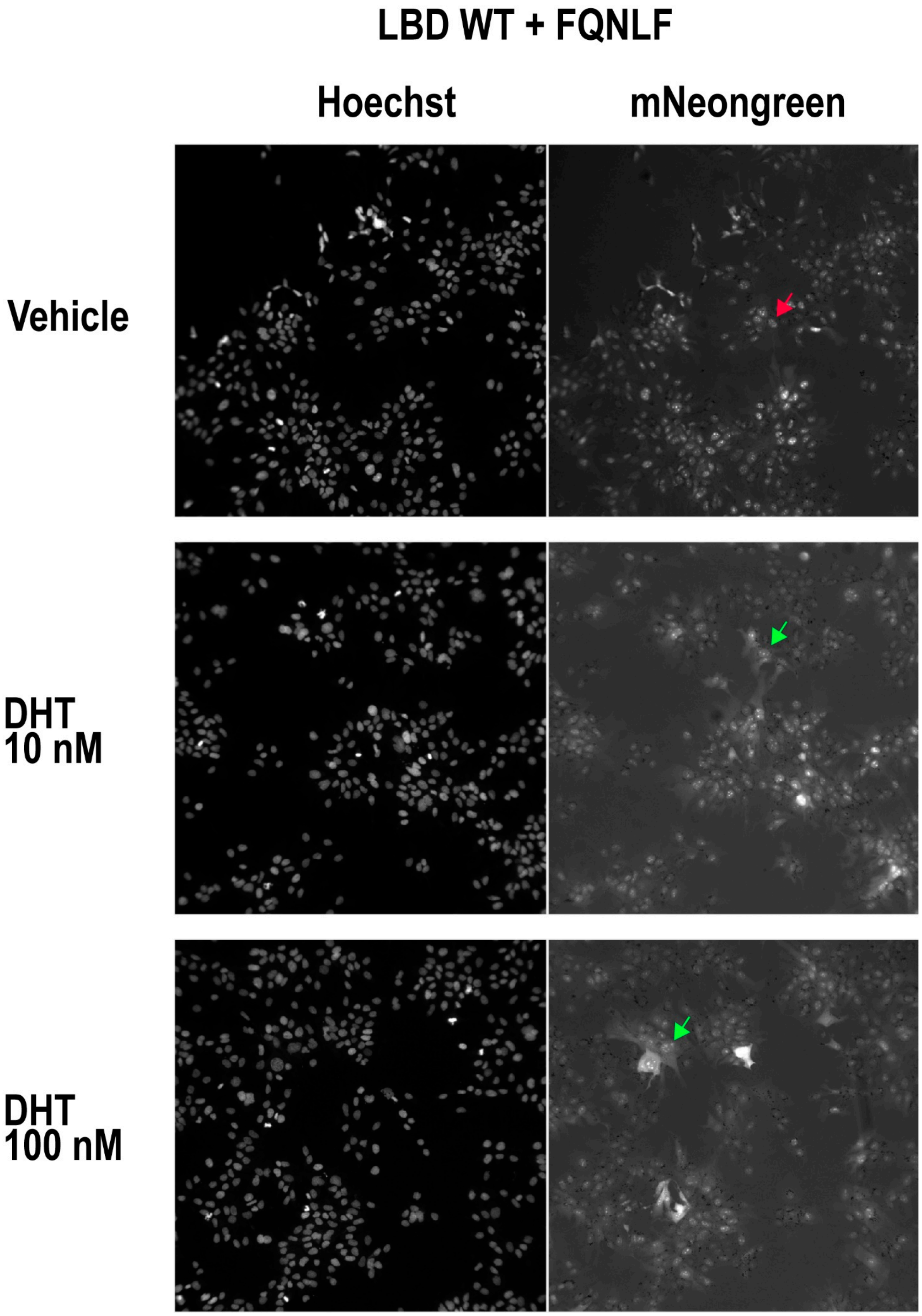

Figure S3: Magnification of split TEV images for LBD WT and FQNLF interaction. Panels on the left show Hoechst staining of the nuclei, while panels on the right represent the signal from the mNeongreen split TEV reporter. Cells with inactive TEV protease (LBD-FQNLF interaction is absent) are indicated with a red arrow, the mNeongreen reporter resides in the nucleus. Cells with active TEV protease (LBD-FQNLF interaction is present) are indicated with a green arrow, the mNeongreen reporter is present in both nucleus and cytoplasm. DHT is required to induce the LBD-FQNLF interaction and is added for overnight incubation at 10 and 100 nM.

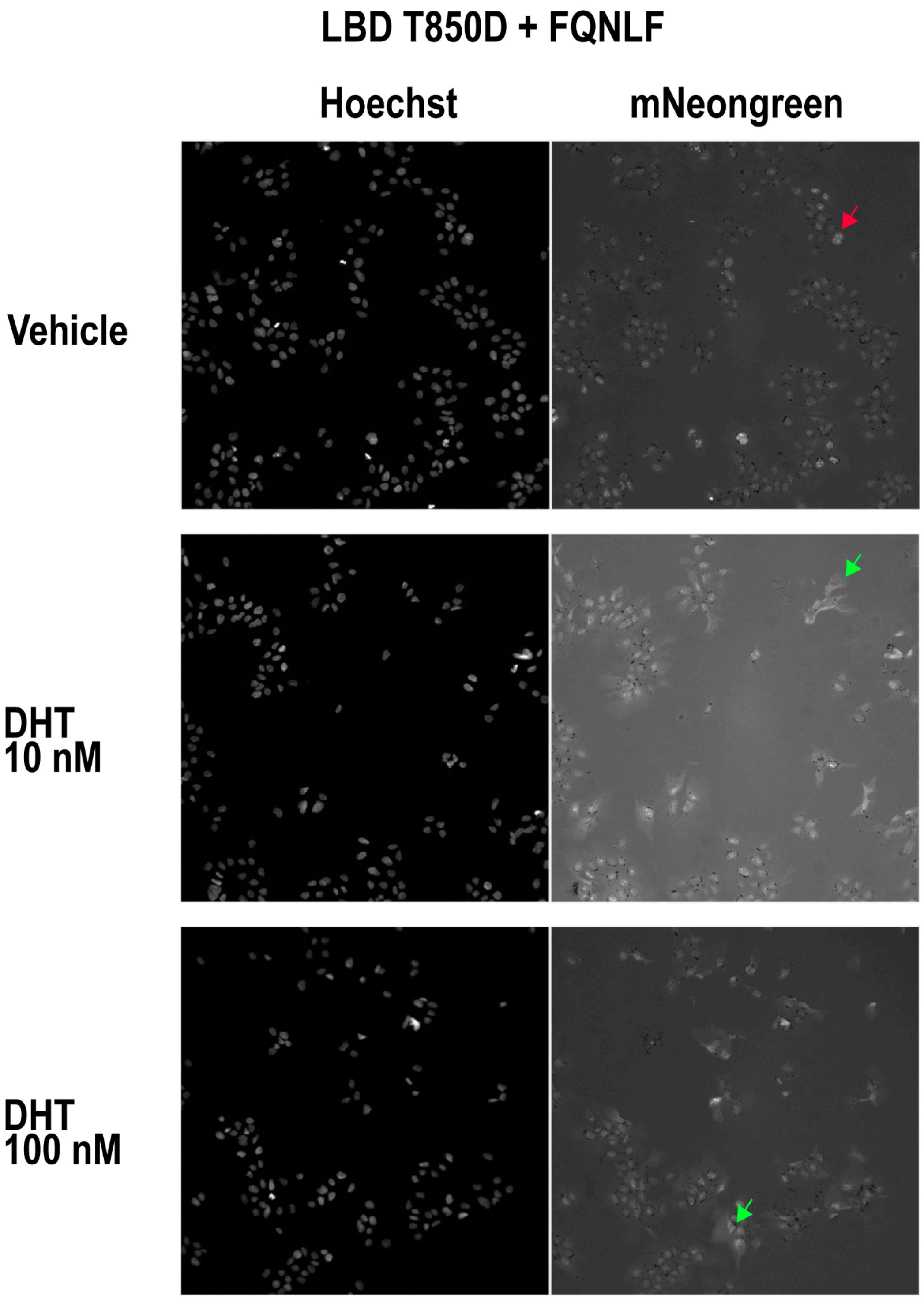

Figure S4: Magnification of split TEV images for LBD T850D and FQNLF interaction. Panels on the left show Hoechst staining of the nuclei, while panels on the right represent the signal from the mNeongreen split TEV reporter. Cells with inactive TEV protease (LBD-FQNLF interaction is absent) are indicated with a red arrow, the mNeongreen reporter resides in the nucleus. Cells with active TEV protease (LBD-FQNLF interaction is present) are indicated with a green arrow, the mNeongreen reporter is present in both nucleus and cytoplasm. DHT is required to induce the LBD-FQNLF interaction and is added for overnight incubation at 10 and 100 nM.

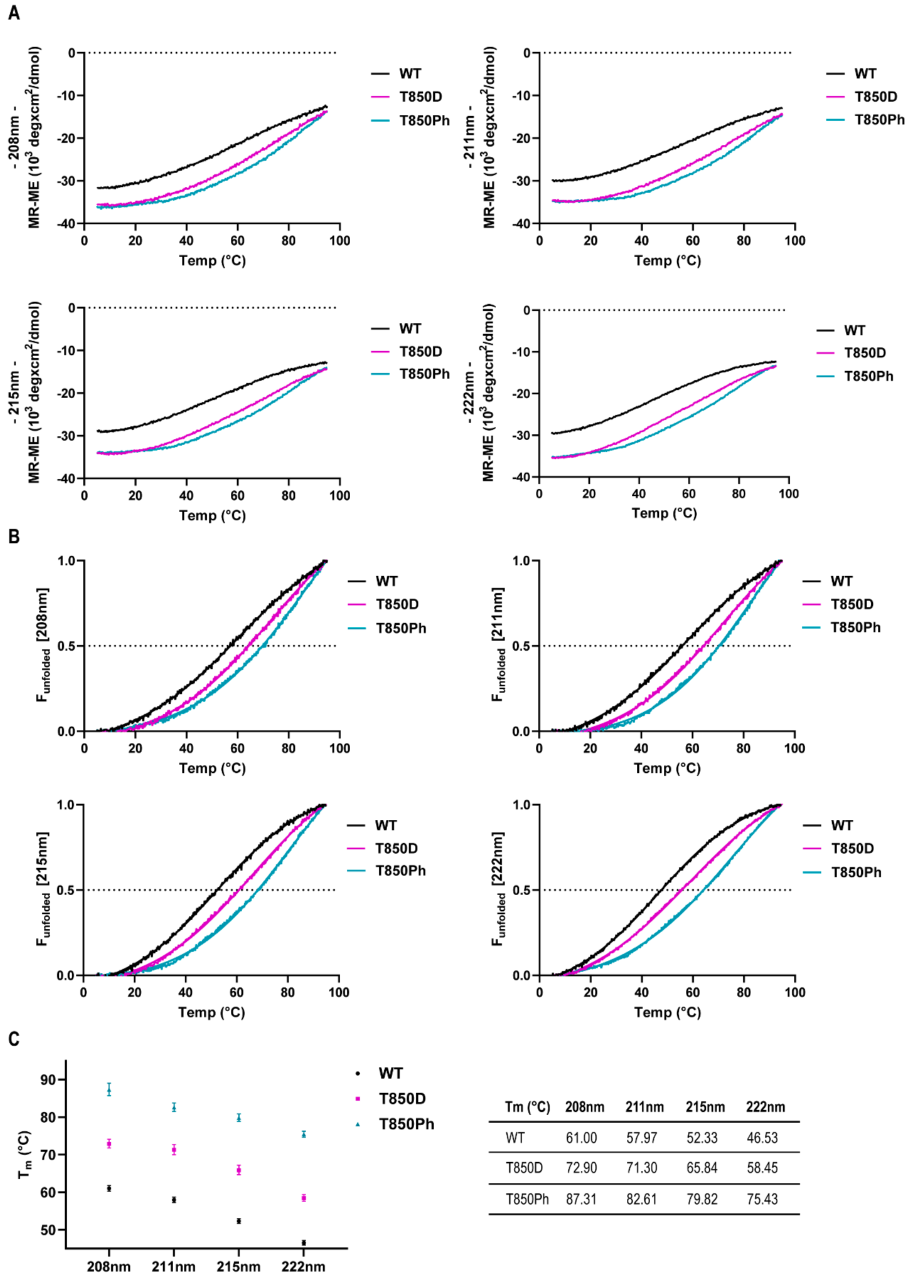

Tm (°C)	208nm	211nm	215nm	222nm
WT	61.00	57.97	52.33	46.53
T850D	72.90	71.30	65.84	58.45
T850Ph	87.31	82.61	79.82	75.43

Figure S5: Thermal denaturation curves of H10 peptides at 208 nm, 211 nm, 215 nm and 222 nm. (A) H10 peptides (aa 845-868 of AR) with T850D or with phosphorylated T850 were compared to WT peptide at different wavelengths during thermal denaturation (5°C-95°C with 0.2°C interval). The CD spectra were taken of peptides at 25µM in 20% TFE-TRIS buffer pH 7.5 (n=2). The MR-ME (Mean Residue-Molar Ellipticity) is given in function of the temperature. (B) The unfolded fraction of the peptide in function of the temperature was calculated with the following equation : $F_{unfolded} = (\theta_t - \theta_{folded})/(\theta_{unfolded} - \theta_{folded})$ with θ_t as the Ellipticity at each temperature, θ_{folded} as the Ellllipticity of the peptide in folded state and $\theta_{unfolded}$ as the Ellipticity of the peptide in unfolded state. The melting temperature (Tm) was calculated via non-linear regression using a sigmoidal dose-response curve with variable slope. The temperature corresponding to an unfolded fraction of 0.5 is the Tm.

(C) Melting temperature (T_m) of H10 peptides (845-868) in °C. The melting points of AR H10 peptides (WT, T850D and T850 Phosphorylated) were determined at different wave lengths: 208 nm, 211 nm, 215 nm and 222 nm. Mean and 95% CI are given of 2 independent experiments.

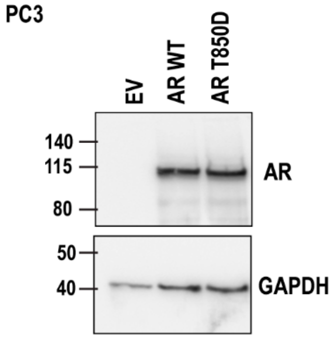

Figure S6: Validation of in-house AR antibody.

The in-house AR antibody generated by the firm Covance, recognizes the aminoterminal part of the AR. For validation of the specificity of the AR antibody, PC3 cells were transiently transfected with empty vector (EV), pSG5-AR WT or pSG5-AR T850D. AR was not detected in the lane transfected with EV. The expected fragment, corresponding to a protein of 110 kDa, appeared in the lanes transfected with AR WT and AR T850D.

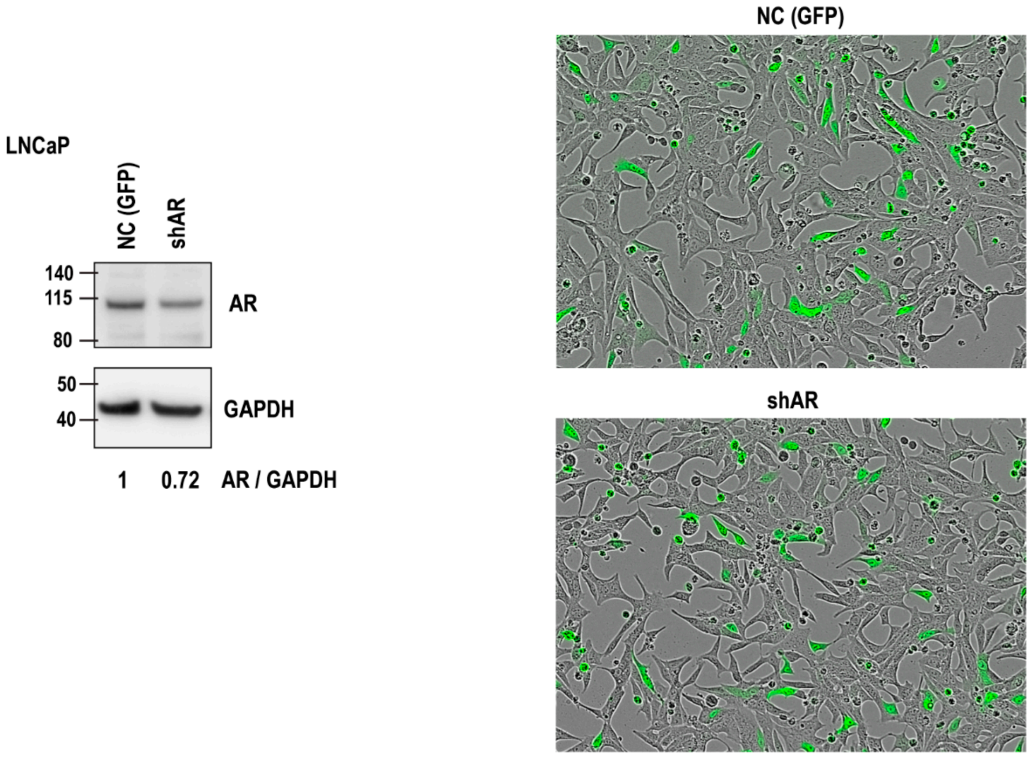

Figure S7: Validation of shAR for knock-down in LNCaP.

To validate the knock-down efficiency of the shAR on the endogenous AR in LNCaP cells, the lentiviral plasmids were transiently transfected via nucleofection using the AMAXA kit R (Lonza). Since both plasmids contain a GFP-expression cassette, transfection efficiency was monitored via the Incucyte Zoom (Essen BioScience). Green Object Confluence was 19% for pCS-CG (Negative control (GFP)) and 23% for pCS-SG shAR (shAR), respectively. Corresponding to the targeting of 23% of LNCaP cells with the pCS-SG shAR plasmid, we observed a reduction of the AR level in the shAR treated LNCaP cells of 28% (AR/GAPDH ratio reduced to 0.72). AR levels were quantified using ImageJ and were normalized by the corresponding GAPDH expression levels.
